# Late Manifestation of Subependymal Giant Cell Astrocytoma With Hydrocephalus in an Adult Patient With Tuberous Sclerosis Complex

**DOI:** 10.7759/cureus.28435

**Published:** 2022-08-26

**Authors:** Sarah Zaher Addeen, Lilyan Bou Yehia, Lubna Aburas, Mhd Firas Safadi

**Affiliations:** 1 Department of Ophthalmology, Al Mouassat University Hospital, Damascus, SYR; 2 Department for Visceral, Thoracic and Vascular Surgery, University Hospital Carl Gustav Carus, Technische Universität Dresden, Dresden, DEU

**Keywords:** hypomelanotic macules, facial angiofibromas, ungal fibroma, hydrocephalus, papilledema, visual acuity, tuberous sclerosis complex, tsc, sega, subependymal giant cell astrocytoma

## Abstract

Subependymal giant cell astrocytoma (SEGA) is a unique brain tumor that constitutes one of the major diagnostic criteria of tuberous sclerosis complex (TSC). It rarely occurs de novo after adolescence. SEGA tends to originate from the ventricular walls, provoking obstructive hydrocephalus, raised intracranial pressure, and papilledema with plausible visual problems. We present a case of large SEGA in a 33-year-old-TSC patient with a higher-than-expected age for the first presentation. His chief complaint was visual acuity deterioration combined with headaches. Microsurgical removal of the tumor was carried out. Obstructive hydrocephalus and papilledema resolved after treatment. Although SEGA-TSC is considered a tumor of children and adolescents, it can present for the first time in adulthood. In TSC patients, periodic imaging follow-up is recommended and any visual symptoms should prompt exclusion of intracranial tumors.

## Introduction

Tuberous sclerosis complex (TSC) is an autosomal dominant hereditary phacomatosis that can affect any organ. The most common presentation of the disease is benign multiple hamartomas that may appear in the brain, heart, kidneys, eyes, and skin [1,2].

The International Tuberous Sclerosis Complex Consensus Group updated the clinical diagnostic criteria for tuberous sclerosis complex in 2012. Subependymal giant cell astrocytoma (SEGA) was classified as a major criterion among others, such as subependymal nodules, cortical tubers, and retinal astrocytoma [1,3]. Isolated pediatric SEGA was scarcely reported in the literature [4,5], as well as in the adult population [6].

SEGA grows on the walls of the lateral ventricles and foramen of Monro, as well as the third ventricle to a less extent [7]. Obstructive hydrocephalus is quite common and nearly universal in childhood. However, silent growth of the tumor with resulting hydrocephalus at a more advanced age was also reported [8].

We present the case of a 33-year-old male with known TSC who presented with visual acuity impairment and headaches because of a large SEGA in the left lateral ventricle with resulting hydrocephalus. The presentation of SEGA for the first time at this age is extremely unusual and has only been reported in a few cases. The case has been reported in line with the SCARE Criteria [9].

## Case presentation

**Figure 1:**
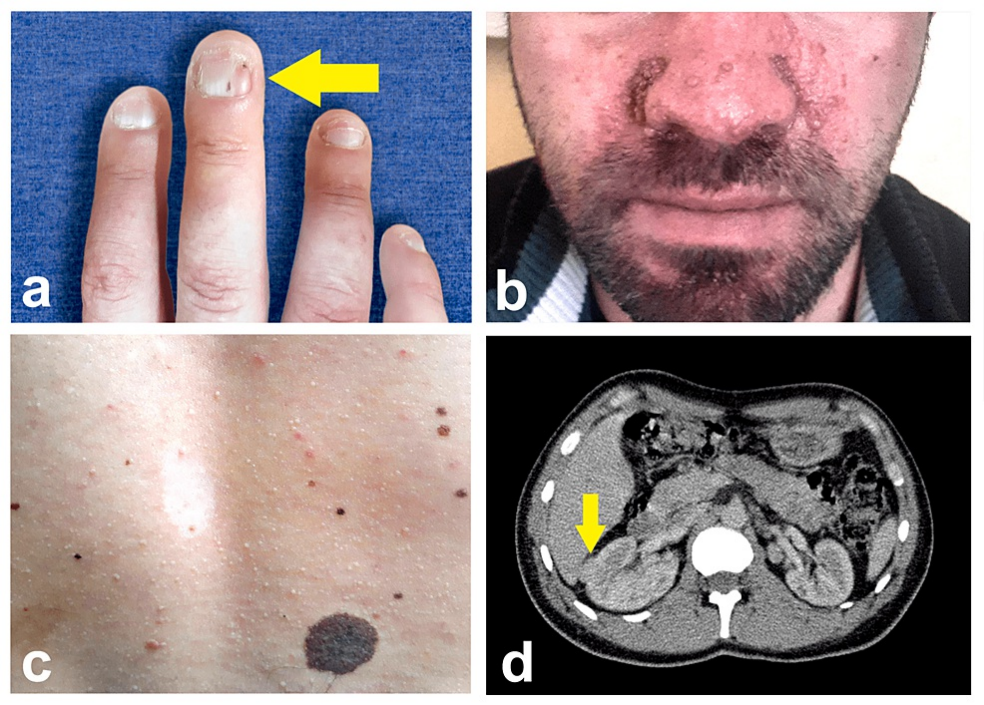
Different manifestations of tuberous sclerosis complex in this case. (a) Ungal fibroma on the middle finger of the right hand (arrow), (b) multiple facial angiofibromas, (c) hypomelanotic macule on the back to the left of the midline, also called ash-leaf spots (the patient displays multiple nevi as well, such as the large one at the bottom-right of the image, which are not part of the syndrome), (d) cortical lesion on the right kidney (arrow).

A 33-year-old male patient presented to the ophthalmologic clinic complaining of gradually deteriorating visual acuity in the left eye over the last several months. He also reported headache episodes of increasing frequency. One year ago, the patient was diagnosed with tuberous sclerosis complex (TSC) based on six major clinical and radiological criteria (Figure 1): ungual fibromas, angiofibromas, hypomelanotic macules, cortical tubers, subependymal nodules, and subependymal giant cell astrocytoma (SEGA). The patient had also known renal lesions as a minor criterion. A family history of TSC was denied.

The initial evaluation revealed an intact visual acuity of 20/20 in the right eye. In the left eye, the visual acuity was low at 20/100 and could be corrected to 20/40. The pupils were round and regular with normal direct and indirect light responses. The fundoscopy revealed papilledema stage I and stage II according to the Frisen staging system in the right and left eyes, respectively. The rest of the ophthalmological assessment, as well as visual fields, was within the normal limits.

**Figure 2:**
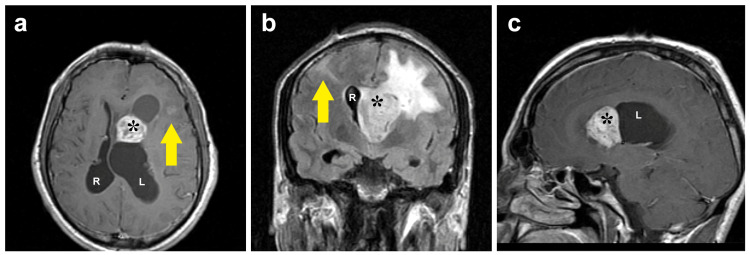
Magnetic resonance imaging of the brain in this case. The images show (a) transverse, (b) coronal, and (c) sagittal sections of the T1-weighted magnetic resonance imaging (MRI) of the brain with gadolinium enhancement showing a 2.5×2.8×3.7 cm lesion on the lateral and frontal wall of the left cerebral ventricle (asterisk). Note also the enlarged left ventricle (L), the midline deviation with the compressed right ventricle (R), and the cortical tubers (arrows).

Magnetic resonance imaging (MRI) with gadolinium enhancement showed cortical tubers, subependymal nodules, and a large, enhancing lesion on the wall of the dilated left lateral ventricle (Figure 2). The lesion was the trigger of obstructive hydrocephalus.

The patient was referred to the neurosurgical department, where surgical removal of the tumor was performed using a left frontal, ultrasound-assisted, transcortical transventricular approach using a microscopic technique. An external ventricular drain was placed before closure. The histological examination confirmed the diagnosis of astrocytoma.

The symptoms resolved at one month postoperatively and there was a significant improvement in the visual acuity as well as papilledema in the left eye. The postoperative MRI after three months showed complete resolution of hydrocephalous. The patient was well after six months.

## Discussion

Tuberous sclerosis complex, also known as Bourneville Pringle disease, was initially described by von Recklinghausen in 1862 [10]. It is an autosomal dominant neurocutaneous phacomatosis, which is associated with benign hamartomas in multiple organ systems [1,2]. The consensus for establishing a definitive, probable, or possible diagnosis was updated in 1998 [11], and was revised later in 2012 by the International Tuberous Sclerosis Complex Consensus Group [3].

The morbidity and mortality associated with this disease are attributed mainly to neurological lesions [12]. Although SEGAs are slow-growth tumors (WHO Grade I) [7], they are responsible for about 25% of mortalities associated with TSC. This percentage levels up to 50% when acute hydrocephalus and intratumor hemorrhage assault [13].

The incidence of SEGA in TSC varies from 5% to 14% [1,2]. SEGA tends to occur in the first and second decades of life. There are also some reports of prenatal and perinatal detection [13]. De novo tumors that arise and manifest for the first time after the age of 25 years are unusual [4,14], which is the case in our 33-year-old patient. Such an unusual occurrence was reported by Adriaensen et al. where the mean age of onset in adults was 31 years (16-58 years) in 43 adults with SEGA from a total of 214 TSC patients [15]. Most other studies included a pediatric population and reported a mean age of 13 years or less [15]. Jansen et al. highlighted the age of patients who presented with SEGA growth and stressed that it is very rare for these tumors to grow beyond the age of 25 even when they present in adulthood. The authors were able to identify only 19 patients (2.4%) with tumor growth after the age of 18 years [14], which emphasizes the rarity of our case.

TSC-SEGA originates mainly in the lateral periventricular regions adjacent to the foramen of Monro [15], and, to a less extent, in the third ventricle [7,16]. As a consequence, they may elicit increased intracranial pressure, seizures, and focal neurological signs [16]. In our case, obstructive hydrocephalus was provoked by the large SEGA in the lateral ventricle which further incited gradual bilateral papilledema one year after the initial diagnosis. This emphasizes the need for regular imaging follow-up in patients with asymptomatic SEGA that are diagnosed in childhood [8,14].

While focal neurological symptoms and headaches are frequent complaints [15], the decline of visual acuity as a chief complaint was only scarcely reported [17]. Other documented manifestations include fatigue, anorexia, increased seizure frequency, cognitive disturbance, sleep disorders, and psychomotor seizures [14,17].

In our patient, surgical intervention was indicated due to the symptomatic obstructive hydrocephalus. Surgery is a safe and effective treatment of SEGA and can be performed using multiple surgical or neuroendoscopic approaches [13] as well as laser interstitial thermal therapy and stereotactic surgery [18,19].

## Conclusions

Subependymal giant cell astrocytoma is a rare, benign tumor of the central nervous system that constitutes a major criterium of the tuberous sclerosis complex. Although it is considered a tumor of children and adolescents, the occurrence and manifestation in later adulthood may still happen. Periodic imaging follow-up is recommended. New complaints in those patients, such as visual symptoms, should prompt thorough investigation with TSC kept in mind.
